# By characterizing metabolic and immune microenvironment reveal potential prognostic markers in the development of colorectal cancer

**DOI:** 10.3389/fbioe.2022.822835

**Published:** 2022-08-05

**Authors:** Liangliang Liao, Yongjian Gao, Jie Su, Ye Feng

**Affiliations:** ^1^ China-Japan Union Hospital of Jilin University, Changchun, China; ^2^ The First Hospital of Jilin University, Changchun, China

**Keywords:** clinical stages, somatic mutation, metabolic and immune, prognostic markers, transcriptional regulation

## Abstract

Colon adenocarcinoma (COAD) is one of the deadliest cancers in the world and survival rates vary significantly between early and advanced stage patients. Therefore, the identification of the pathogenesis in the development of COAD and prognostic markers is urgently demanded. Herein, we collected RNA-seq and somatic mutation data of COAD for statistical analysis. Clinical stage-specific differentially expressed genes (DEGs) and tumor development-dependent DEGs were identified. By characterizing the metabolic and immune features of COAD between stages, we found that the energy supply and inflammatory response of advanced tumors were suppressed. Next, the *ETS1, AR, GATA1, GATA2, SREBF1, FOXP3, STAT4,* and *NFKB1* were identified to drive the metabolic and immune-related pathways in the development of COAD. The three potential prognostic markers (*HOXC8, IRF7,* and *CXCL13*) were identified based on Cox regression analysis. Additionally, immune infiltration analysis revealed that the resting CD4^+^ T cell was significantly related to the overall survival (OS) of COAD patients. Collectively, the specific metabolic and immune characteristics of advanced patients and the identified prognostic biomarkers will contribute to the development of precision medicine.

## Introduction

Colorectal cancer is one of the deadliest cancers in the world, killing nearly a million people every year (Labianca et al., 2010; Dekker et al., 2019). Although advances in diagnosis and treatment methods improve the prognosis of early-stage patients, it is still an important cause of cancer-related deaths (Lech et al., 2016). The locations where tumors often occur are divided into proximal colon, distal colon, and rectum, and the ratio of patients reaches 4:2:3 (Cheng et al., 2011). With the development of diagnostic technology, the number of young patients diagnosed with colon cancer increase. Recent study data suggest that the overall five-year relative survival rate for COAD patients exceeds 60%, it varies depending on the clinical stages. Therefore, the dynamic changes in the physiological mechanisms involved in the development of COAD need to be urgently determined.

In the process of tumor development, it is accompanied by metabolic reprogramming to support tumor cells’ demand for proliferation and metastasis (DeBerardinis and Chandel, 2016). Genetics and environmental are important driving factors of cell metabolism (Boroughs and DeBerardinis, 2015; Pavlova and Thompson, 2016). Among them, different tumor stages have specific physiological environments (La Vecchia and Sebastian, 2020). To explore metabolic reprogramming during tumor development, it is necessary to use gene expression to measure metabolic pathway activity (Puram et al., 2017).

So far, the diagnosis of colorectal cancer still relies on colonoscopy, but the treatment methods have developed significantly. In recent years, immune checkpoint therapy have become hot spots in cancer treatment. For example, a novel treatment for colorectal cancer was proposed based on the immune checkpoint PD-1/PD-L1 (Yaghoubi et al., 2019). However, not all COAD patients show complete response to PD-1, and there are some adverse reactions (Wu et al., 2019). Hence, in this work, we explored the immune cell landscape and the activity of immune-related pathways in the development of COAD.

In this study, we collected RNA-seq and mutation data of colorectal tumors and normal samples from The Cancer Genome Atlas (TCGA) database to identify metabolic and immune characteristics in the development of COAD. Transcriptional regulatory networks were constructed to identify drive factors that play important roles in immune and metabolic pathways. Potential prognostic markers identified by Cox regression analysis were used to construct survival risk models for COAD. Moreover, immune infiltration analysis revealed the immune landscape of COAD.

## Materials and methods

### Data collection

First, we downloaded the gene expression data (including tumor samples and normal samples), somatic mutation data and clinical information of COAD patients from the TCGA database (Tomczak et al., 2015). The hallmark, KEGG, GO Biological Process and metabolic pathway gene sets were collected from The Molecular Signatures Database (MSigDB (Liberzon et al., 2015), http://www.gsea-msigdb.org/gsea/msigdb/) database. Further, the transcription factors (TF)-TG data for human were downloaded from the TRRUST (Han et al., 2018) (https://www.grnpedia.org/trrust/) and ORTI (Vafaee et al., 2016) databases (http://orti.sydney.edu.au/about.html). The signature profiles of leukocyte were collected from the CIBERSORTx (Newman et al., 2015) (https://cibersortx.stanford.edu/) database.

### Differential expression analysis

Here, in addition to considering the difference in gene expression between all the tumor sample and the normal sample collected from TCGA, the difference in gene expression between the tumor sample and the corresponding normal sample in each clinical stage was also considered. Differentially expressed genes were identified using the R package Limma (Ritchie et al., 2015). We considered the genes with |*log*_*2*_ *FC*| > 1.5 and *p-value* < 0.01 as the differentially expressed RNAs (DEGs).

### Statistical analysis of mutation data

The somatic mutation data of COAD collected from TCGA was used to describe the mutation of signature genes. Then, the R package maftools (Mayakonda et al., 2018) was used for the statistical and visualization of mutation location, mutation form, mutation frequency and other information for these signature genes.

### Gene set level analysis

For the metabolic gene sets collected from the MSigDB database, gene set variation analysis (Hanzelmann et al., 2013) (GSVA) was used to calculated the enrichment score of each stage of COAD in metabolic pathway by R package GSVA (v1.36.3). GSVA is a non-parametric, unsupervised algorithm. Further, the ten pathways and biological pathways related to immune checkpoints, antigen presentation, and immune activation or suppression were extracted from hallmark, KEGG and GO Biological Process gene sets. Using the appealed GSVA algorithm, the activity score of each stage of COAD in these immune-related pathways was calculated.

### Construction of transcriptional regulatory network

First, we used the analysis of variance (ANOVA) algorithm to calculate the metabolic genes and immune-related genes specifically expressed between samples grouped in stages. We defined the metabolic and immune-related DEGs specifically expressed between samples grouped by stage as stage-MDEGs and stage-IDEGs. Next, stage-MDEGs and stage-IDEGs were extracted for the construction of transcriptional regulatory network based on the TF-target gene data collected from TRRUST and ORTI databases. The TF-target gene relationship pairs related to stage-MDEGs and stage-IDEGs were extracted. Further, the Pearson correlation coefficient (*R*) between the genes of each pair was calculated and the cutoff of the *p*-value and *R* were set to 0.05 and 0.2. Moreover, the TFs-target genes network was constructed using Cytoscape (Shannon et al., 2003) (3.7.0) tool. The topological properties of the network then were calculated and the top genes of degree were identified as key drive factors.

### Functional enrichment analysis

First, the genes whose expression was affected through the transcriptional regulatory mechanism was collected. The R package clusterprofiler (Yu et al., 2012) was used to perform GO functional enrichment on these genes. We set *p*-value<0.05 to screen for significantly enriched biological pathway. The relationship between these biological pathways and the corresponding genes was depicted using the R package circlize (Gu et al., 2014) (v0.4.10).

### Identification of prognosis markers

Based on the appealed transcriptional regulatory network research, the TF and target genes were used as candidate factors for the identification of prognostic markers of COAD. First, the univariate COX regression was used to screen for the prognostic related genes using patient’s survival data including survival state and overall survival (OS) of COAD (the cutoff of *p-value* was 0.05). The patients of COAD were randomly sub-grouped into the training set and test set in accordance with the ratio of 7:3. Further, the train set were used to construct multivariate COX regression model (Fisher and Lin, 1999). The reliability of the survival prediction model was described by the receiver operating characteristic curve (ROC), and the area under the curve (AUC) was calculated. The PH hypothesis test was also used to calibrate the model. The gene that *p*-value of the Schoenfeld Individual test greater than 0.05 was reserved for the reconstruction of the multivariate cox regression model. Moreover, we used the nomogram algorithm to build a COAD survival risk prediction model.

### Calculation of risk score

First, we calculate the risk score of each patient for COAD based on the linear combination of the expression values weighted by the coefficients of the multivariate Cox regression analysis:

$$Risk\;score\;\left(i\right)=\sum_{k=1}^{n}\beta_{k}*e_{ki}$$ (1)

Where n represents the number of prognostic-related genes, i represents the order of genes, and k represents the order of patients. The regression coefficient and gene expression value are represented by β and e respectively. Then, we calculated the risk scores of the samples and divided the samples into high-risk and low-risk categories based on the median risk score. The Kaplan-Meier survival curve (Ranstam and Cook, 2017) was used to describe the patient’s survival probability of high- and low-risk group, and calculated the statistical difference with the bilateral log-rank test (Guyot et al., 2012). Besides, the above survival analysis process was also carried out in an independent data set (GSE38832 (Tripathi et al., 2014)) to confirm the robustness and stability of prognostic markers.

## Results

### Stage-specific transcriptional and mutational landscape in Colon adenocarcinoma development

**FIGURE 1:**
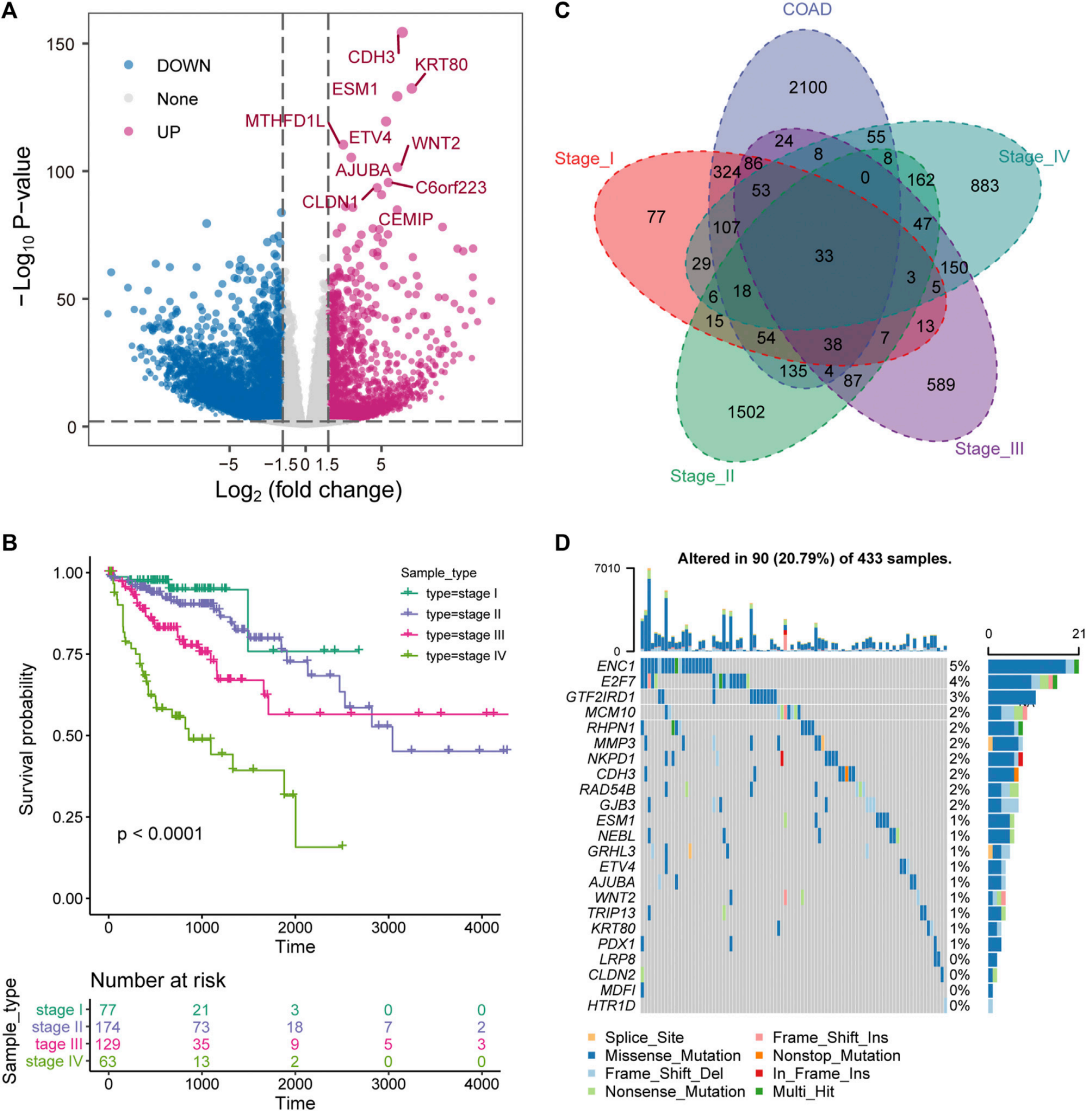
Stage-specific transcriptional and mutational landscape in COAD development. **(A)** The results of the DEGs between the whole tumor sample of COAD and the normal sample are displayed by volcano plot. The x-axis represents log2 (Fold Change). The y-axis displays -log10 (*p* value). **(B)** Kaplan-Meier (KM) curves depict the survival of patients in four stages for COAD. Log-rank test is used to calculate statistical significance. **(C)** Venn diagram shows the intersection of up-regulated genes between clinical stages and overall tumor samples. **(D)** The waterfall chart shows the mutation information of genes that are continuously up-regulated in the development of COAD, and the mutation type of each gene in each sample is displayed.

The key to the treatment of colorectal cancer is early detection and timely diagnosis (The, 2018). Therefore, exploring the dynamic changes of molecules in the development of COAD was beneficial to reveal the driving mechanism of the physiological state of patients in different stages. We developed a pipeline to explore the dynamic molecular mechanisms in COAD development (Supplementary Figure S1). First, differential expression analysis revealed that 9,859 DEGs (3,047 up-regulation and 6,812 down-regulation) were identified between tumor samples and para-cancerous samples of COAD (Figure 1A). The top 10 up-regulated genes were marked. Among them, *WNT2* is an important component in the WNT signaling pathway and promotes tumor angiogenesis in colon cancer (Unterleuthner et al., 2020). With the development of tumors, the prognosis of advanced patients will be severely disrupted (Huang et al., 2020), which was also effective for COAD. We found that the prognosis of COAD patients is consistent with its clinical stage and that advanced patients were associated with the worst OS (Figure 1B). Further, to explore the specific expression of biomolecules in patients of four clinical stages, we have statistically tested the DEGs of each clinical stage. The 2326 DEGs (868 up-regulation and 1,461 down-regulation) in stage I, the 7,857 DEGs (1,959 up-regulation and 5,898 down-regulation) in stage II, the 3,976 DEGs (1,147 up-regulation and 2,820 down-regulation) in stage III, and the 4,200 DEGs (1,320 up-regulation and 2,880 down-regulation) in stage IV were identified. By integrating the DEGs identified in the overall tumor sample and the DEGs identified in each clinical stage, 33 up-regulated genes and 117 down-regulated genes were identified in different stages (Figure 1C), which indicated that molecular and functions have been reprogrammed during the development of COAD. For the 33 up-regulated genes, we used COAD mutation data to describe their mutation landscape (Figure 1D). Among them, according to the current mutation data, there are no somatic mutations in the genomic positions of 10 genes. We found that ENC1 has the top mutation frequency (Figure 1D), and it was significant co-occurence with *GRHL3, RHPN1* and *E2F7* at the mutation level (Supplementary Figure S2). Moreover, previous studies have shown that *ENC1* promotes the progression of colorectal cancer through *JAK2/STAT5/AKT* axis-mediated epithelial-mesenchymal transition and stemness (Cui et al., 2021).

### Stage-specific metabolic and immune activity

**FIGURE 2:**
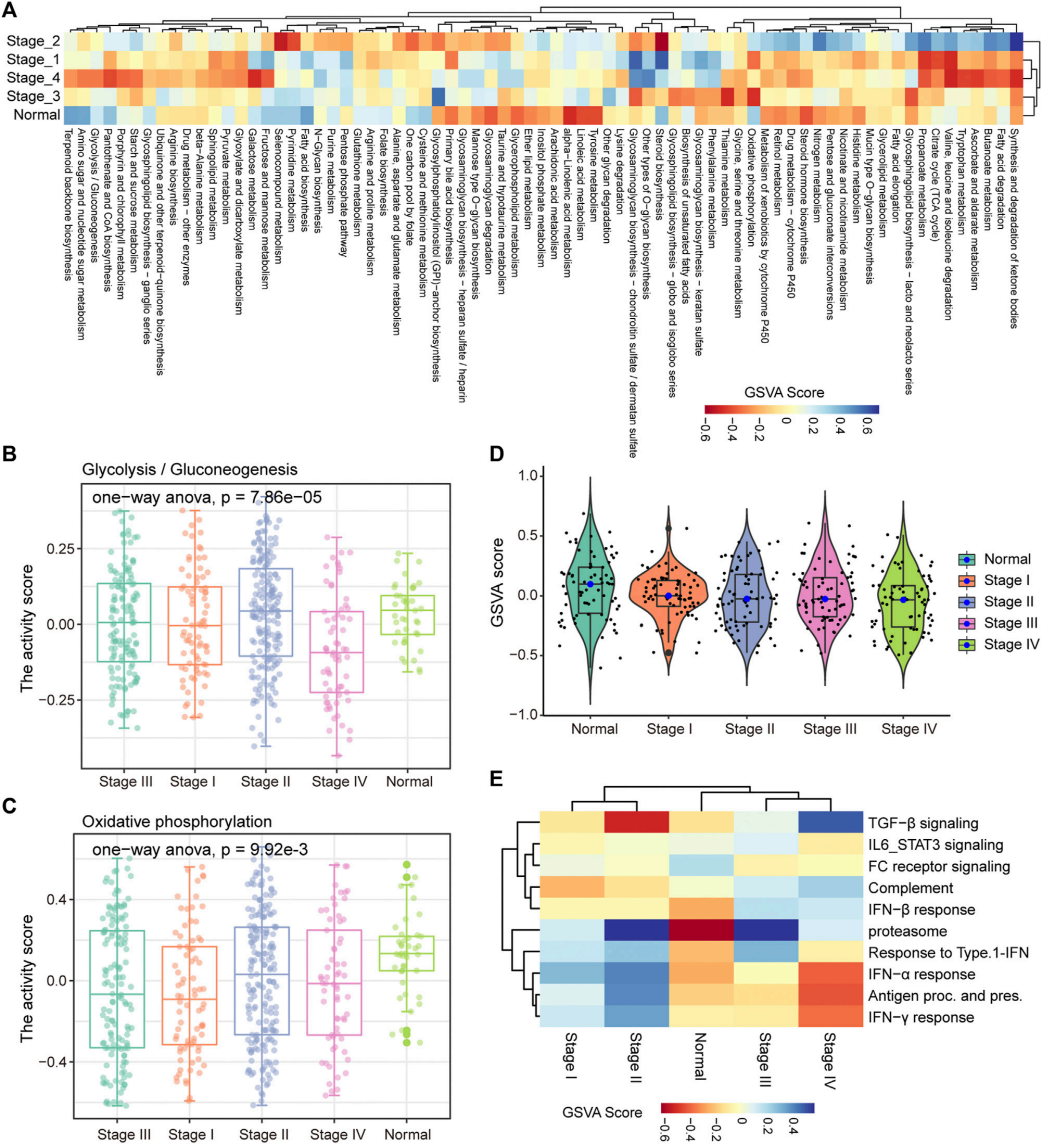
Stage-specific metabolic and immune activity. **(A)** The enrichment scores of the 85 metabolic pathways calculated by GSVA in the five categories (stage I, stage II, stage III, stage IV, normal samples) are displayed by the heat map. **(B–C)** Boxplot shows the glycolysis and OXPHOS pathway scores of each sample in the five categories. ANOVA is used to assess statistical differences between groups. **(D)** The distribution of enrichment scores for 85 metabolic pathways in the five categories is shown by violin chart. **(E)** The heat map shows the enrichment scores of each immune-related pathway in the five categories.

In the process of tumor development, tumor cells undergo metabolic reprogramming to adapt to changes of the environment (Sun et al., 2018). To characterize the dynamic changes of metabolism in the development of COAD, GSVA was used to calculate the activity of 85 metabolic pathways collected from the MsigDB database in four clinical stages. We found that tumor tissue have activated energy supply compared with normal tissues (Figure 2A), which was consistent with previous studies showing that the activated metabolic microenvironment could supply tumor proliferation and metastasis (Wang et al., 2021). In different stages of tumor development, there are obvious differences in the activity of metabolic pathways. The activities of glycolysis and oxidative phosphorylation (OXPHOS) have been significantly enhanced in stage III (Figures 2B,C), which may be related to the proliferation of tumor cells at this stage. Furthermore, we analyzed the activity distribution of metabolic pathways between normal tissues and tumors of various stages. The global metabolic activity of normal tissues was higher than that of tumor tissues of each stage (Figure 2D), which indicates that tumor cells could selectively activate specific metabolic pathways to adapt to the development of this stage. For example, tumors in stage IV have high purity and metastasis to distant organs (Koo et al., 2020), and the reduction of glycolysis and the enhancement of OXPHOS were important metabolic characteristics of this stage for COAD (Figures 2B,C), which may be related to the increase in oxygen supply caused by the formation of blood vessels in local tissues. With the development of tumors, the immune microenvironment of tumors has also changed. We found that there were significant differences in the activity of the immune signaling pathways between the early and late stages of COAD (Figure 2E). In stage I and II of COAD, IL6/STAT3 signaling pathway, TGF-β signaling and FC receptor response have strong activity, which revealed the inflammatory activation of the immune response in the early stage of the tumor. Taken together, these results suggested that the development of COAD was accompanied by metabolic reprogramming and variation of the immune microenvironment.

### Key factors drive the reprogramming of metabolism and immunity

**FIGURE 3:**
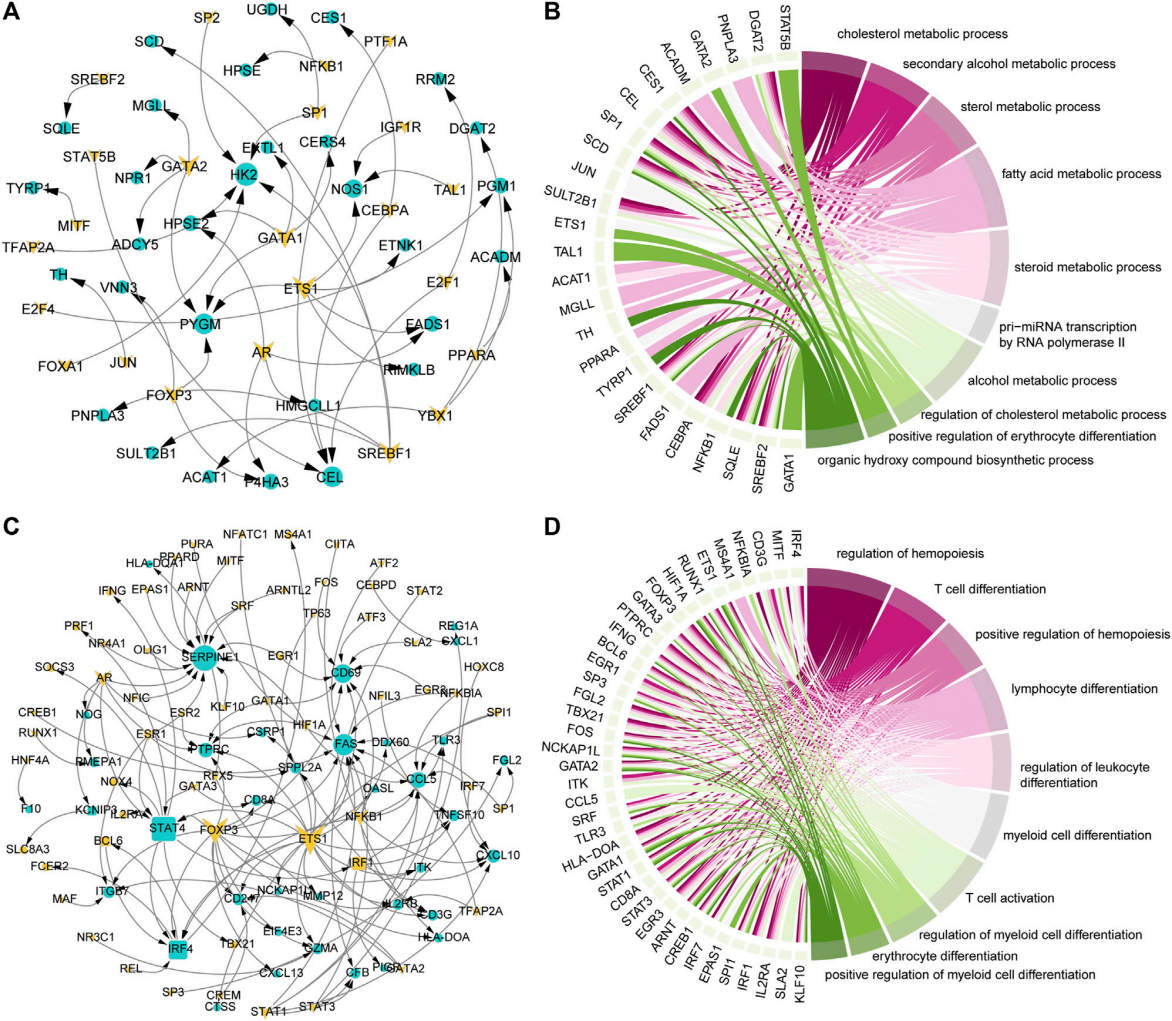
Key factors drive the reprogramming of metabolism and immunity. **(A)** Transcriptional regulatory network of metabolism-related genes that continue to be specifically expressed in the development of COAD. The triangle represents TF, the circle represents the target gene. Genes that continue to be specifically expressed in the development of COAD are marked in yellow. **(B)** The top 10 GO items enriched by genes in the **(A)** network. The interaction between genes and the GO items is shown. **(C)** Same as in **(A)** but for immune-related genes that continue to be specifically expressed in the development of COAD. **(D)** Same as in **(B)** but for genes in the **(C)** network.

TFs play an important role in gene expression by regulating the initiation and intensity of gene transcription (Lambert et al., 2018). To identify the driving factors that regulate metabolism and immune activity in the development of COAD, the transcriptional regulatory networks were constructed. Based on metabolism-related genes, 23 TFs and 30 target genes were identified and 46 TF-target gene units were formed (Figure 3A). By analyzing the topological properties of the network, we have identified the top 5 TFs (*ETS1, AR, GATA1, GATA2,* and *SREBF1*) of degree. The *ETS1* has been shown to be a driving factor for the progression of majority cancers (Dittmer, 2015; Chen et al., 2019) and its down-regulation inhibits the progression of colorectal cancer (Gu et al., 2019). These results indicated that *ETS1, AR, GATA1, GATA2,* and *SREBF1* could be used as biomarkers in the development of COAD. In addition to metabolic pathways, the results of functional enrichment analysis showed that genes involved in the transcriptional regulatory network were significantly enriched in the transcription of non-coding RNA and cell differentiation (Figure 3B). In the immune-related transcriptional regulatory network, 55 TFs and 48 target genes constituted the 129 TF-target gene units (Figure 3C). The *ETS1* also the top gene of degree in this network. The remaining four high degree TFs were *FOXP3, STAT4, AR,* and *NFKB1.* Among them, the *FOXP3* was closely related to the differentiation of T cells and was lineage-defining TF for regulatory T cells (Ono, 2020). Moreover, we found that the genes in the immune-related transcriptional regulatory network were significantly enriched in the activation and differentiation of immune cells (Figure 3D). Taken together, these results suggested that the *ETS1* and *AR* were the driving factors of metabolic and immune reprogramming in the development of COAD.

### Potential prognostic signature for Colon adenocarcinoma

**FIGURE 4:**
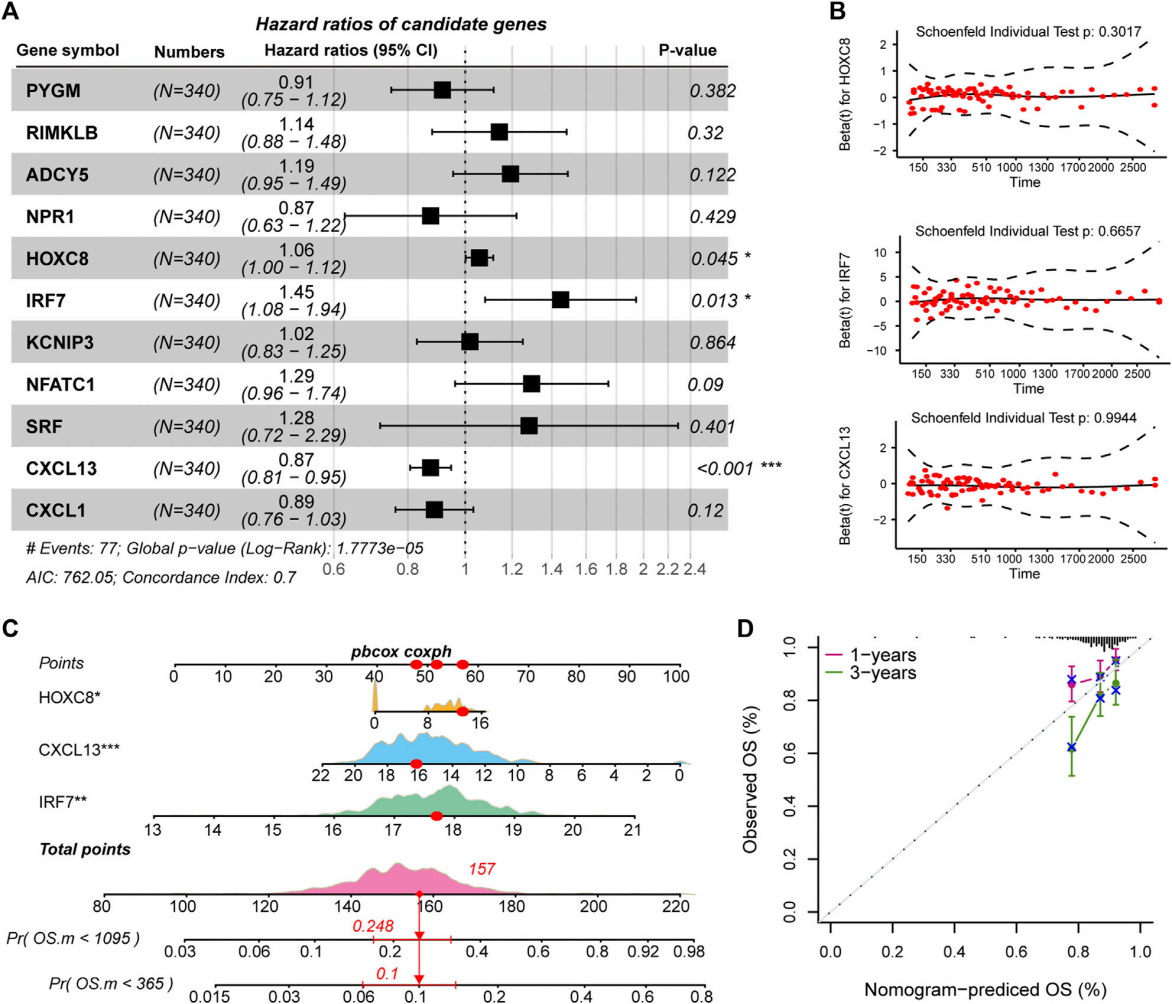
Identification of prognostic related genes in COAD. **(A)** Forest plots for multivariate Cox risk regression models. **(B)** PH hypothesis test of *HOXC8, IRF7,* and *CXCL13.* x-axis represents survival time, and y-axis represents Schoenfeld residuals. **(C)** The nomogram shows the prediction of 1-year and 3-years survival risk for patients of COAD. **(D)** Calibration curve of nomogram.

**FIGURE 5:**
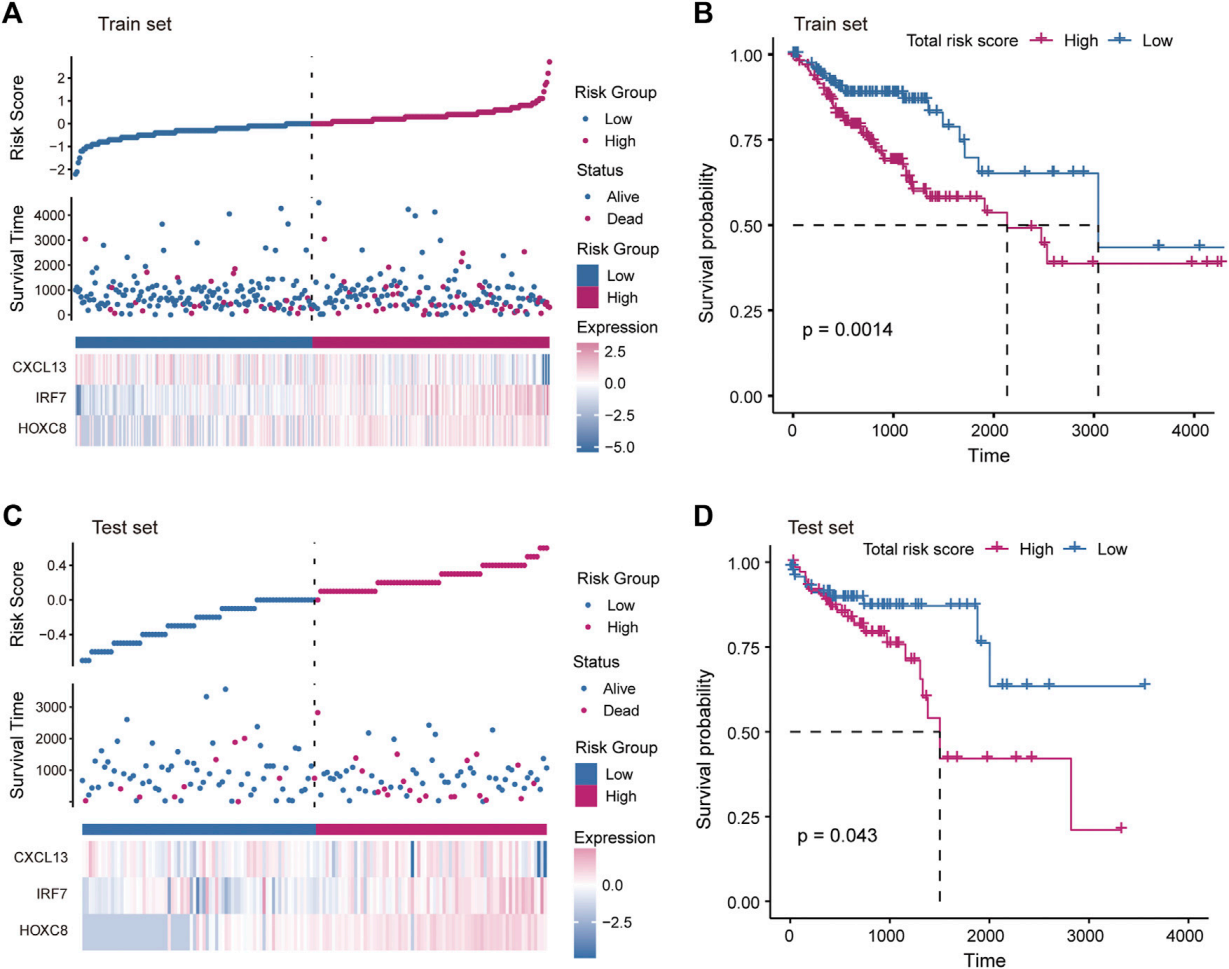
Construction of risk scoring model. **(A)** The figure shows the risk scores, survival status, and expression of prognostic markers for the train set samples. **(B)** The Kaplan-Meier curves for the survival of high-risk and low-risk groups in the train set. **(C)** Same as in **(A)** but for the test set samples. **(D)** Same as in **(B)** but for two groups in the test set.

**FIGURE 6:**
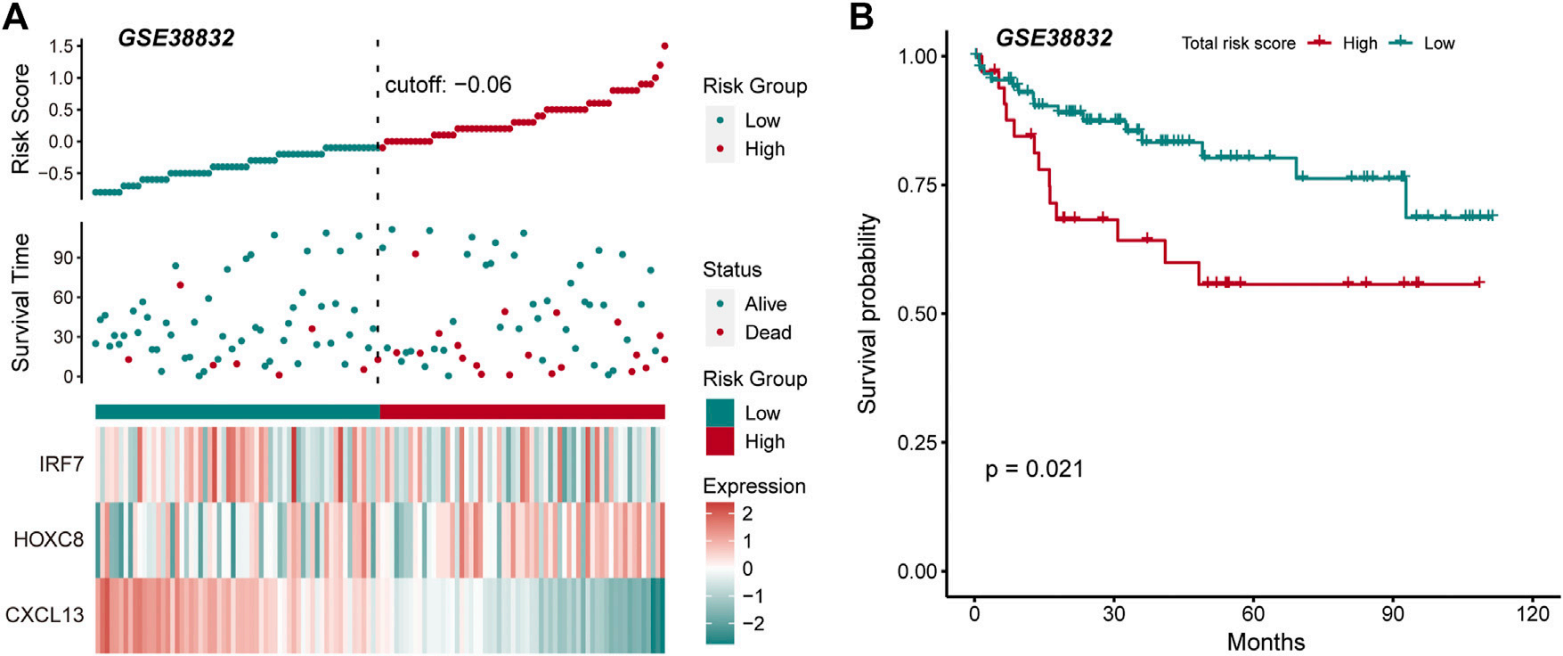
External data verify the robustness and stability of prognostic markers. **(A)** The figure shows the risk scores, survival status, and expression of prognostic markers for the GSE38832 set. **(B)** The Kaplan-Meier curves for the survival of high-risk and low-risk groups in the GSE38832 set.

The driving factors that play an important role in the development of COAD by regulating cell metabolism and immunity may determine the prognosis of patients. To identify potential prognostic markers of COAD, we integrated the statistical analysis pipeline from previous studies in this work (Dang et al., 2021; Le et al., 2021). The univariate Cox regression algorithm was used to fit the relationship between gene expression and patient’s survival based on train set (including survival status and survival time). In this step, 11 genes were identified and significantly related to the patient’s OS. Further, these genes were used to construct the multivariate Cox regression model. We found that three genes including *HOXC8, IRF7,* and *CXCL13* could be used as a potential prognostic signature for COAD (Figures 4A,B). To identify the best predictive time point for the survival prediction model, we divided the 1–3 years period into four periods and evaluated the prediction results using receiver operating characteristic curve (ROC). We found that the risk prediction result reached the maximum area under curve (AUC) value of 0.69 in the 912.5 days (Supplementary Figure S3). Based on these three prognostic markers, 1-year and 3-years survival risk prediction models of COAD were constructed and visualized through the nomograph (Figure 4C). The results of the calibration curve proved the stability of the risk prediction model (Figure 4D). Moreover, the risk scoring model was constructed as follows: risk score = -0.06 * *HOXC8* + 0.37* *IRF7* -0.13* *CXCL13*. The risk scores of the training and test set samples were calculated and they were divided into high risk and low risk groups based on the median risk score. We found the obvious expression difference of the three prognostic signature between the high/low-risk groups (Figure 5A) and the patients of high-risk score had poor prognosis (Figure 5B). The test set also showed the same prediction results as the train set (Figures 5C,D). Moreover, in the set of GSE38832 series, 122 samples were divided into two groups according to the upper quartile of risk scores (Figure 6A). Similarly, patients with high-risk scores had poorer OS (Figure 6B). All these suggesting that the *HOXC8, IRF7,* and *CXCL13* contributed to the prediction of the patient’s prognosis for COAD and could be used for clinical diagnosis.

### Immune cell components relate to the patient’s survival risk

**FIGURE 7:**
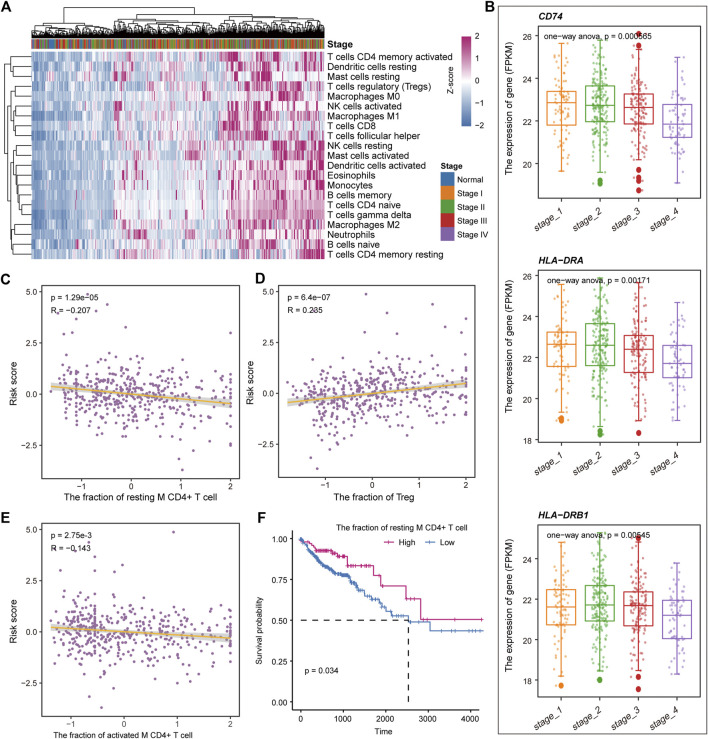
Immune cell components relate to the patient’s survival risk. **(A)** The immune cell composition of each sample is displayed by heat map. The column label represents the clinical stage of the sample. **(B)** The expression levels of genes encoding MHC II molecules in each clinical stage are shown by boxplot. ANOVA is used to calculate statistical significance. **(C–E)** The correlation between the risk score and the fraction of the resting CD4^+^ T cell, activated M CD4^+^ T cell, and Treg. **(F)** Kaplan-Meier curves for survival in high/low-fraction groups of the resting CD4^+^ T cell. Log-rank test was used to calculate statistical significance.

Previous studies have shown that the immune microenvironment plays an important role in the development of tumors (Hinshaw and Shevde, 2019; Lei et al., 2020). To identify the immune characteristics in the development of COAD, the CIBERSORTx tool was used to calculate the immune cell composition of samples for COAD and normal. For the 22 immune cell fraction matrices obtained, we found that COAD patients had immune infiltration compared with normal samples and there was no significant difference in immune infiltration between the clinical stages of tumors (Figure 7A). Further, the content of major histocompatibility complex (MHC) was calculated. We found that the gene encoding MHC-II molecule has a lower expression level in Stage IV, but a higher expression level in stage I and II (Figure 7B), which may explain the loss of immunogenicity in advanced patients of CAOD. Moreover, we evaluate the correlation of the immune cell fraction and risk score for patients of COAD. We found that the fraction of resting CD4^+^ T cell, activated M CD4^+^ T cell, and Treg were significantly related to the patient’s survival risk (Figures 7C–E). Based on the median of each immune cell component, the patients of COAD were divided into two groups (high/low-fraction). For the resting CD4^+^ T cell, the patients with low-fraction of resting CD4^+^ T cell were related to poor patient’s prognosis (Figure 7F), suggesting that resting CD4^+^ T cell may be a protective factor for COAD. Taken together, all these indicate that immune infiltration and tumor immunogenicity were closely related to the development and patient’s survival of COAD.

## Discussion

In this work, we have revealed the metabolic and immune characteristics in the development of COAD by integrating multi-omics data analysis. We found that COAD patients with different clinical stages had significant prognostic differences and advanced patients had the worst prognosis. For each clinical stage, stage-specific genes are identified and integrated analysis reveals 33 up-regulated genes and 117 down-regulated genes in all clinical stages. Combined with the somatic mutation data of the patients, the mutation landscape of these genes in COAD was revealed. Furthermore, stage-specific metabolic and immune activity were revealed through functional enrichment analysis. We found that energy metabolism (including glycolysis and OXPHOS) contributed to the development of COAD and is the basis for the changes in the physiological mechanism of each clinical stage. By constructing transcriptional regulatory networks, we have identified the key factors driving the development of COAD by disturbing metabolic and immune pathways. Moreover, we have identified three prognostic markers (*HOXC8, IRF7,* and *CXCL13*) of COAD based on the Cox regression algorithm and constructed a risk score model for the assessment of patient survival risk. By combining the patient’s immune infiltration and survival data, we found that the resting CD4^+^ T cell can be used as a protective factor for the patient.

Colorectal cancer is the fourth most deadly cancer in the world, causing nearly 900,000 deaths each year (Dekker et al., 2019). Since the disease only has symptoms in the late stages, it is necessary to identify its development mechanism and potential biomarkers. In recent years, there were majority studies on biomarkers and prognostic markers of COAD (Pellino et al., 2018; Patel et al., 2019). For example, Razi et al. revealed DCLK1 as a marker of stem cell regulates tumor progression and invasion from the perspective of ceRNA mechanism (Razi et al., 2021). Pankaj Ahluwalia et al. simply used KM analysis and Cox regression algorithm to identify prognostic markers of COAD (Ahluwalia et al., 2019). We focused on the development of tumors and were committed to revealing its dynamic physiological mechanisms. The clinical stage of COAD patients was revealed to be significantly related to the prognosis, indicating that the clinical stage could partly reflect the development of the tumor.

In the transcriptional regulatory network, we have identified hub TFs for metabolism and immune regulation of COAD. The *ETS1* and *AR* were the driving factors both of metabolic and immune pathway, suggesting that *ETS1* and *AR* could be used as potential biomarkers for COAD. We found that patients with COAD have global immune cell infiltration compared with normal tissues and the wide heterogeneity of immune cells in each clinical stage, which is consistent with previous studies (Ge et al., 2019).

## Conclusion

In summary, our research revealed the metabolic and immune characteristics in the development of COAD, and identified potential biomarkers through biological network analysis. Three potential prognostic markers were identified. Through immune infiltration analysis, the immune landscape of COAD was revealed and the resting CD4^+^ T cell was identified as a protective factor.

## Data Availability

The original contributions presented in the study are included in the article/Supplementary Material, further inquiries can be directed to the corresponding author.
