# Supplementary material for: By characterizing metabolic and immune microenvironment reveal potential prognostic markers in the development of colorectal cancer
Source: Front Bioeng Biotechnol. 2022 Aug 5;10:822835. doi: 10.3389/fbioe.2022.822835 (PMC9390973; doi:10.3389/fbioe.2022.822835)
Supplement: Supplementary file 1 [file Table1.DOCX]

**Supplementary Figures**

**
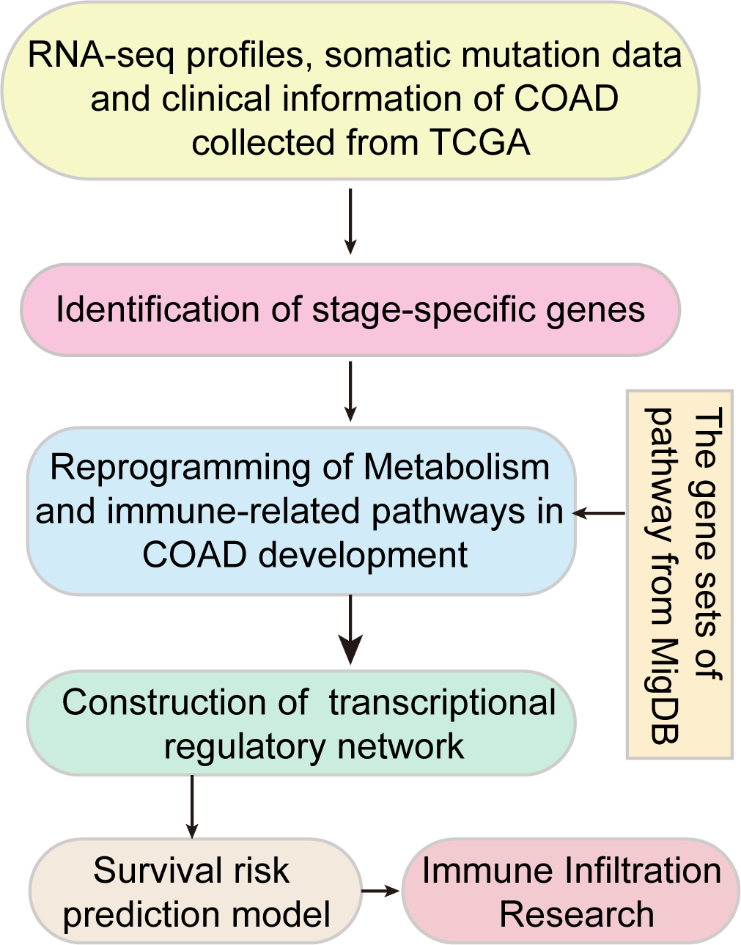
**

**Figure S1. Flowchart showed the content of this work.**

**
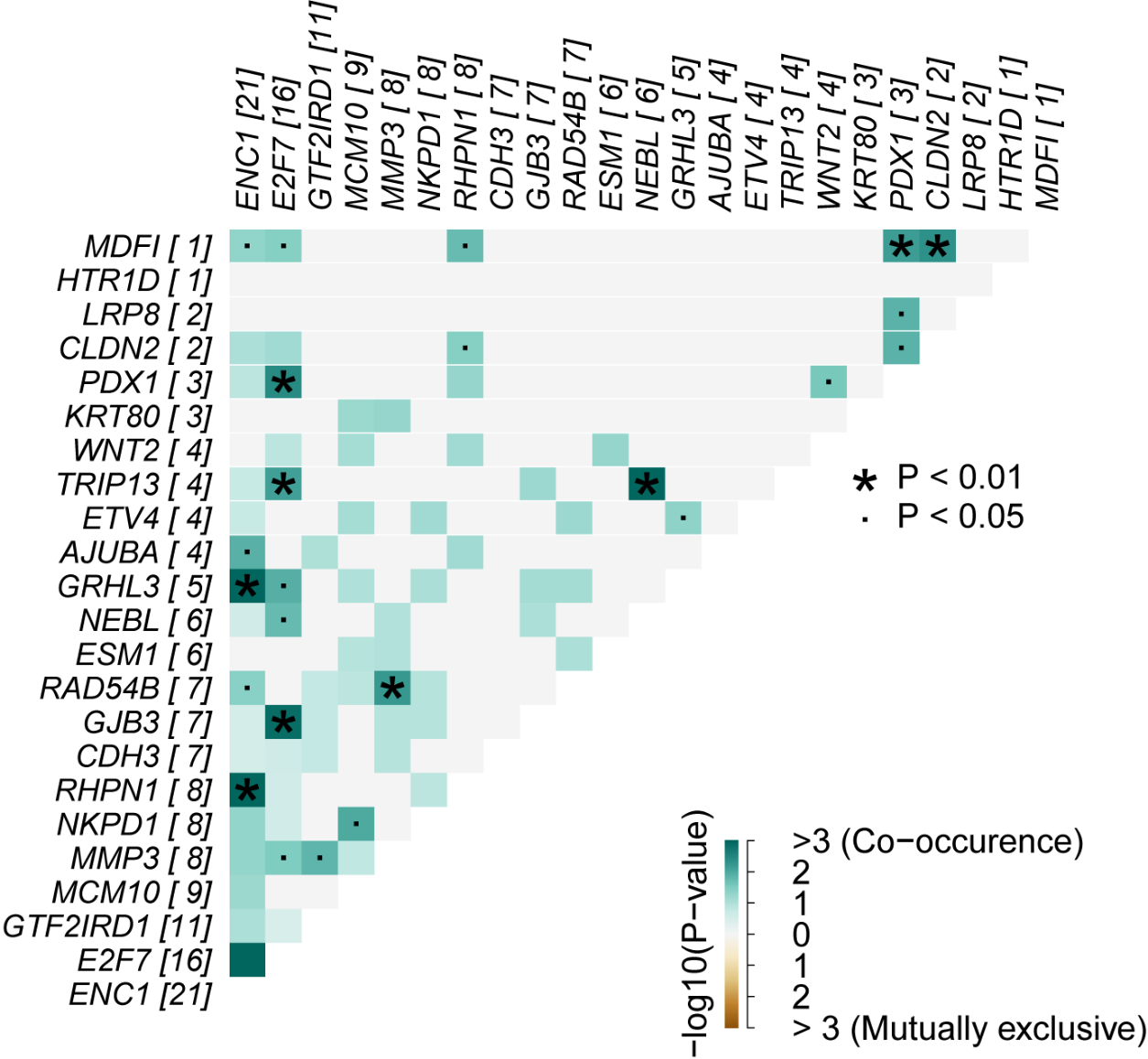
**

**Figure S2.** Mutation correlation heatmap of genes that are continuously up-regulated in the development of COAD. Locations with significant correlations are marked by stars.


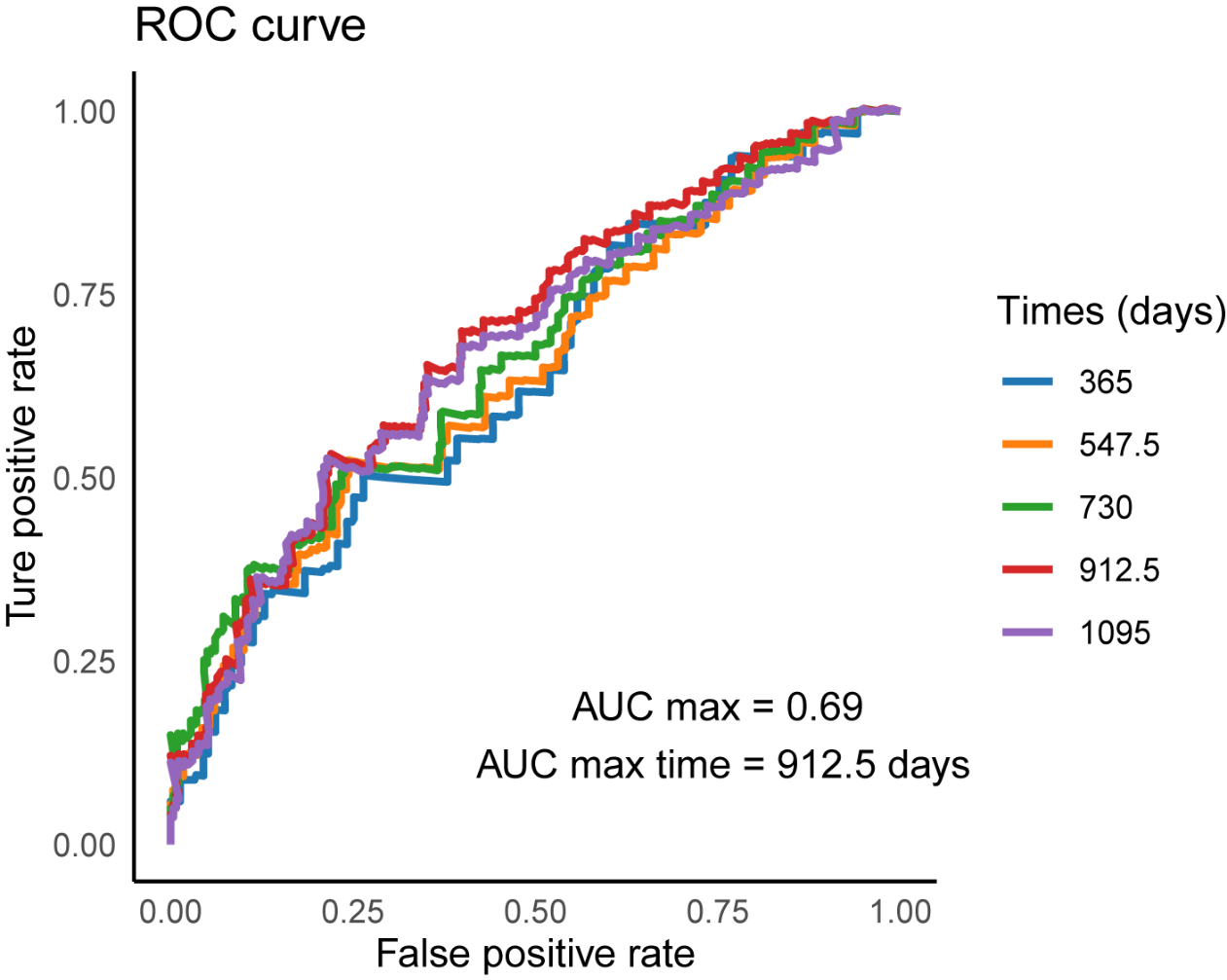


**Figure S3.** The ROC curve reflects the predictive power of the risk regression model at five time points. The different colored curves represent specific time-points.
