# Supplementary material for: By characterizing metabolic and immune microenvironment reveal potential prognostic markers in the development of colorectal cancer
Source: Front Bioeng Biotechnol. 2022 Aug 5;10:822835. doi: 10.3389/fbioe.2022.822835 (PMC9390973; doi:10.3389/fbioe.2022.822835)
Supplement: Supplementary file 2 [file Table2.DOCX]

**Reply to Referee #1**

*This study proposes a comprehensive bioinformatics analysis to characterize metabolic and immune microenvironment reveal potential prognostic markers in the development of colorectal cancer. Although the idea is of interest and fits the journal scope, some major points should be addressed:*

1. *It is important to validate the findings on an external dataset or via experimental studies.*

**REPLY:** We thank the referee for this comment. Your comments is of great value to the improvement of this work. This study was mainly based on statistical analysis of biological multi-omics data. Among the identified prognostic markers, we collected the GSE38832 series from the GEO database to validate the reliability of the risk score model. The method description of this section in the Methods section of the manuscript as follow:

**Page 8, Line 11**

Besides, the above survival analysis process was also carried out in an independent data set (GSE38832 [1]) to confirm the robustness and stability of prognostic markers.

2. *There are a lot of grammatical errors and typos in the manuscript. Thus, the authors are suggested to re-check and revise carefully in the revision.*

**REPLY:** We thank the referee for this comment. There are indeed multiple grammatical errors, improper wording and editing errors in the manuscript. Your question would help improve the manuscript. To address your comment, we invited the professional assistance of several colleagues to review and revise our manuscript.

3. *ROC curve and AUC analysis should be conducted in survival analysis.*

**REPLY:** We thank the referee for this comment. Your comment is scientific and professional. We plotted ROC curves to assess the predictive power of survival risk models. We have revised the manuscript and figure as follow:

**Page 7, Line 16**

The reliability of the survival prediction model was described by the receiver operating characteristic curve (ROC), and the area under the curve (AUC) was calculated.

**Page 13, Line 6**

To identify the best predictive time point for the survival prediction model, we divided the 1 to 3 years period into four periods and evaluated the prediction results using receiver operating characteristic curve (ROC). We found that the risk prediction result reached the maximum area under curve (AUC) value of 0.69 in the 912.5 day (Supplementary Figure S3).

**Figure S2.** The ROC curve reflects the predictive power of the risk regression model at five time points. The different colored curves represent specific time-points.

*4. Source codes should be provided for replicating the studies.*

**REPLY:** We thank the referee for this comment. We have sorted out the Source codes and uploaded them together in this reply.

*5. The combination of different analyses i.e., multivariate analysis, KM plot, risk score, ... is well-known so that the authors should provide more references to attract a broader readership such as PMID: 34572330, PMID: 34298828.*

**REPLY:** We thank the referee for this comment. Your suggestion has been fully considered and additional references have been added to appeal to a wider readership. We have revised the manuscript as follow:

**Page 12, Line 21**

To identify potential prognostic markers of COAD, we integrated the statistical analysis pipeline from previous studies in this work [2; 3].

*6. The authors should provide more related works in literature review on bioinformatics-based studies on COAD or specific biomarkers.*

**REPLY:** We thank the referee for this comment. We review previous work on biomarker identification for COAD, and discuss it in the Discussion section of the manuscript. We have revised the manuscript as follow:

**Page 16, Line 22**

In recent years, there were majority studies on biomarkers and prognostic markers of COAD [4; 5]. For example, Razi et al. revealed DCLK1 as a marker of stem cell regulates tumor progression and invasion from the perspective of ceRNA mechanism [6]. Pankaj Ahluwalia et al. simply used KM analysis and Cox regression algorithm to identify prognostic markers of COAD [7].

*7. Equations should be numbered.*

**REPLY:** We thank the referee for this comment. We added the corresponding numbered for equations. We have revised the manuscript as follow:

**Page 8, Line 3**

Cox regression analysis:

$Risk score \left( i \right)=\sum_{k=1}^{n} \beta_{k}*e_{ki}$	(1)

8. *There must have space before reference number.*

**REPLY:** We thank the referee for this comment. In the process of re-reviewing the manuscript, we have standardized the use of spaces before reference numbers. For example:

**Page 9, Line 3**

The key to the treatment of colorectal cancer is early detection and timely diagnosis [8].

We sincerely appreciate the positive feedback from the reviewer. We believed that we have addressed the reviewer’s comment. Please don't hesitate to contact us if you have any further questions.

**Reference:**

[1] M.K. Tripathi, N.G. Deane, J. Zhu, H. An, S. Mima, X. Wang, S. Padmanabhan, Z. Shi, N. Prodduturi, K.K. Ciombor, X. Chen, M.K. Washington, B. Zhang, and R.D. Beauchamp, Nuclear factor of activated T-cell activity is associated with metastatic capacity in colon cancer. Cancer Res 74 (2014) 6947-57.

[2] H.H. Dang, H.D.K. Ta, T.T.T. Nguyen, G. Anuraga, C.Y. Wang, K.H. Lee, and N.Q.K. Le, Identifying GPSM Family Members as Potential Biomarkers in Breast Cancer: A Comprehensive Bioinformatics Analysis. Biomedicines 9 (2021).

[3] V.H. Le, Q.H. Kha, T.N.K. Hung, and N.Q.K. Le, Risk Score Generated from CT-Based Radiomics Signatures for Overall Survival Prediction in Non-Small Cell Lung Cancer. Cancers (Basel) 13 (2021).

[4] J.N. Patel, M.K. Fong, and M. Jagosky, Colorectal Cancer Biomarkers in the Era of Personalized Medicine. J Pers Med 9 (2019).

[5] G. Pellino, G. Gallo, P. Pallante, R. Capasso, A. De Stefano, I. Maretto, U. Malapelle, S. Qiu, S. Nikolaou, A. Barina, G. Clerico, A. Reginelli, A. Giuliani, G. Sciaudone, C. Kontovounisios, L. Brunese, M. Trompetto, and F. Selvaggi, Noninvasive Biomarkers of Colorectal Cancer: Role in Diagnosis and Personalised Treatment Perspectives. Gastroenterol Res Pract 2018 (2018) 2397863.

[6] S. Razi, A. Sadeghi, Z. Asadi-Lari, K.J. Tam, E. Kalantari, and Z. Madjd, DCLK1, a promising colorectal cancer stem cell marker, regulates tumor progression and invasion through miR-137 and miR-15a dependent manner. Clin Exp Med 21 (2021) 139-147.

[7] P. Ahluwalia, A.K. Mondal, C. Bloomer, S. Fulzele, K. Jones, S. Ananth, G.K. Gahlay, S. Heneidi, A.M. Rojiani, V. Kota, and R. Kolhe, Identification and Clinical Validation of a Novel 4 Gene-Signature with Prognostic Utility in Colorectal Cancer. Int J Mol Sci 20 (2019).

[8] H. The Lancet Gastroenterology, Colorectal cancer screening: is earlier better? Lancet Gastroenterol Hepatol 3 (2018) 519.
