# Supplementary material for: By characterizing metabolic and immune microenvironment reveal potential prognostic markers in the development of colorectal cancer
Source: Front Bioeng Biotechnol. 2022 Aug 5;10:822835. doi: 10.3389/fbioe.2022.822835 (PMC9390973; doi:10.3389/fbioe.2022.822835)
Supplement: Supplementary file 3 [file Table3.DOCX]

**Reply to Referee #3**

*The work by Feng et al. reveals some interesting and key point aspects about metabolic and immune characteristics in the development of COAD. Through their detailed and discrete analysis, the authors were able to identify potential biomarkers through biological network analysis, in particular three potential prognostic markers were identified along with a specific protective factor.*

*The analysis was conducted through a well known specific pipeline and procedure. However, I would suggest the authors to include in their manuscript a specific figure or sketch summarising and listing the entire step-by-step approach they applied, in order to provide to the audience and to the scientific community the stepwise approach they adopted and explain how they obtain their results.
I would also require to the authors to address the following typos and check the entire manuscript clearing any eventual misprints:
1- Then ,the R package maftools -> Then, the R package maftools
2- Moreover, The TFs-target genes -> Moreover, the TFs-target genes
3- Further, we analyzed -> Furthermore, we analyzed
4- Further, stage-specific metabolic-> Furthermore, stage-specific metabolic*

**REPLY:** We thank the referee for this comment. We draw a flowchart to summarize the general idea and analysis process of this study. We have revised the manuscript and figure as follow:

**Page 9, Line 6**

We developed a pipeline to explore the dynamic molecular mechanisms in COAD development (Supplementary Figure S1).

**Figure S1. Flowchart showed the content of this work.**

Furthermore, we also invited the professional assistance of several colleagues to review and revise our manuscript. The typos of your proposed were corrected in the manuscript as follows:

**Page 5, Line 21**

Then, the R package maftools [1] was used for the statistical and visualization of mutation location, mutation form, mutation frequency and other information for these signature genes.

**Page 6, Line 21**

Moreover, the TFs-target genes network was constructed using Cytoscape [2] (3.7.0) tool.

**Page 11, Line 1**

Furthermore, we analyzed the activity distribution of metabolic pathways between normal tissues and tumors of various stages.

**Page 16, Line 9**

Furthermore, stage-specific metabolic and immune activity were revealed through functional enrichment analysis.

We sincerely appreciate the positive feedback from the reviewer. We believed that we have addressed the reviewer’s comment. Please don't hesitate to contact us if you have any further questions.

**Reference:**

[1] A. Mayakonda, D.C. Lin, Y. Assenov, C. Plass, and H.P. Koeffler, Maftools: efficient and comprehensive analysis of somatic variants in cancer. Genome Res 28 (2018) 1747-1756.

[2] P. Shannon, A. Markiel, O. Ozier, N.S. Baliga, J.T. Wang, D. Ramage, N. Amin, B. Schwikowski, and T. Ideker, Cytoscape: a software environment for integrated models of biomolecular interaction networks. Genome Res 13 (2003) 2498-504.
